# Implications of standardization of serum 25-hydroxyvitamin D data for the evaluation of vitamin D status in Germany, including a temporal analysis

**DOI:** 10.1186/s12889-018-5769-y

**Published:** 2018-07-06

**Authors:** Martina Rabenberg, Christa Scheidt-Nave, Markus A. Busch, Michael Thamm, Nina Rieckmann, Ramón A. Durazo-Arvizu, Kirsten G. Dowling, Zuzana Škrabáková, Kevin D. Cashman, Christopher T. Sempos, Gert B. M. Mensink

**Affiliations:** 1 Department of Epidemiology and Health Monitoring, Robert Koch Institute, General-Pape-Straße 62-66, 12101 Berlin, Germany; 2 Institute of Public Health, Charité–Universitätsmedizin, Seestraße 73, 13347 Berlin, Germany; 3 Department of Public Health Sciences, Stritch School of Medicine, Loyola University Chicago, Maywood, IL 60153 USA; 4 Cork Centre for Vitamin D and Nutrition Research, School of Food and Nutritional Sciences, University College Cork, College Road, Cork, T12 K8AF Ireland; 5 Vitamin D Standardization Program (VDSP), 520 Ferdinand Dr, Havre de Grace, MD 21078 USA

**Keywords:** Vitamin D, 25(OH)D, Vitamin D deficiency, Standardization, Comparability, Population survey, Germany

## Abstract

**Background:**

Comparability of 25-hydroxyvitamin D (25(OH)D) measurements is hampered by method-related differences in measurement values. International standardization of laboratory assays has been suggested to solve this problem.

**Methods:**

As part of the European Commission–funded project ‘Food-based solutions for optimal vitamin D nutrition and health through the life cycle’ (ODIN), original measurements of serum 25(OH)D of three German national health surveys conducted between 1998 and 2011 have been standardized retrospectively. In these representative population-based samples including persons aged between 1 and 79 years, the original 25(OH)D values were compared with those after standardization. Mean values and prevalences of vitamin D deficiency, insufficiency, and sufficiency (25(OH)D levels < 30, 30- < 50, and > =50 nmol/l, respectively) were calculated by sex and age groups based on original and standardized 25(OH)D data.

**Results:**

In comparison to the original 25(OH)D levels, the standardized levels showed higher means overall and in age- and sex-specific analyses. After standardization, the prevalence of vitamin D deficiency was lower in all surveys while the prevalence of vitamin D sufficiency was higher. Nevertheless, even after standardization ~ 15% of adults and 12.5% of children had serum 25(OH)D levels < 30 nmol/l. Thus, the proportion of deficient vitamin D levels in the German population is still considerable.

**Conclusions:**

The use of standardization of 25(OH)D levels has a substantial impact on estimates of the vitamin D status in Germany. Since clinical diagnostic, therapeutic and public health decision-making require valid and comparable data, standardization and calibration of commercial, clinical and research laboratory assays for 25(OH)D measurement should become common practice. Until then, researchers, health practitioners and policy makers should be aware of the peculiarities of the measurement methods when comparing and interpreting 25(OH)D levels.

## Background

In the last decade, there has been an explosion of research related to, as well as major public interest in, the health impacts of vitamin D. A long-recognized endocrine function of vitamin D is the regulation of calcium and phosphorus metabolism. As vitamin D plays an important role in the mineralization of bone, it is not surprising that long-term deficiency can lead to metabolic bone disorders, including rickets in children and osteomalacia or osteoporosis in adults [1–5]. In addition, there have been numerous reports of associations between vitamin D status with a wide spectrum of health conditions and diseases beyond bone, including diabetes mellitus, cardiovascular diseases and different types of cancer [2, 6, 7], even though much about the causal pathway(s) involved is still unclear.

To assess vitamin D status, 25(OH)D measured in serum or plasma is an accepted indicator and widely used in both epidemiological research and clinical practice [8]. However, for the measurement of 25(OH)D, different assays are available including competitive binding-protein assays, immunoassays (e.g. chemiluminescent immunoassay [CLIA]), high performance liquid chromatography (HPLC), and liquid chromatography-tandem mass spectrometry (LC-MS/MS), which is currently considered to be more accurate and precise [9, 10]. Several studies have shown that different laboratory procedures can yield markedly divergent results for the measured 25(OH)D values due to inter- and between-assay variability as well as inter- and between-laboratory discrepancies [11–14]. Those widespread, method-related differences in results of total 25(OH)D hamper comparability of 25(OH)D measurements and progress in the field of vitamin D and health. In particular, they confound the comparison of vitamin D status between countries and World regions as well as assessment of temporal changes. Accordingly, there has been efforts in recent times to standardize the measurement of circulating 25(OH)D in both clinical and research laboratories [15, 16].

The Vitamin D Standardization Program (VDSP) organized by the Office of Dietary Supplements of the National Institutes of Health, USA, was established in November 2010 to address the issue [11, 15, 16]. The main goal of the VDSP is the promotion of a standardized 25(OH)D measurement which is accurate and comparable between different time points, laboratories, and laboratory procedures by calibration of commercial, clinical and research laboratory 25(OH)D assays. A principal objective is the standardization of 25(OH)D measurement in national health and nutrition surveys by applying VDSP standardization protocols [11]. The VDSP protocols for standardization of serum 25(OH)D data from past surveys have been applied to national surveys in Canada [17], the US [18] and a number of nationally or regionally representative samples in Europe [16, 19]. As part of the European standardization exercises, two German national health surveys (‘German Health Interview and Examination Survey for Adults’, DEGS1, and ‘German Health Interview and Examination Survey for Children and Adolescents’, KiGGS) [20–22] were included in the wider collection of 14 European population studies (*n* = 55,844) [23]. However, this exercise only reported data on serum 25(OH)D on the entire population. While these analyses permit the estimation of standardized 25(OH)D levels and propensities of vitamin D deficiency in the contributing countries, they, by design, do not provide a deeper insight into potential differences among age-groups within the wider population groups.

In the present work, we used this opportunity to provide a more in-depth analysis of vitamin D status and to compare prevalences of vitamin D deficiency, insufficiency and sufficiency in the German population based on the original measurements with those after standardization from the two surveys but also stratified by age-groups. In addition, we used standardized serum 25(OH)D data from an older German national health survey of adults (‘German National Health Interview and Examination Survey 1998’, GNHIES98), not previously reported, to undertake a comparison of data from two cross-sectional surveys in Germany over a ten year period. This new data is of special interest for clinical practice and public health policy.

## Methods

### Study design and subjects

GNHIES98, DEGS1, and KiGGS were conducted by the Robert Koch Institute, Berlin. The design and methods have been described in detail elsewhere [24–30]. In brief, the sample design of each survey included two steps. First, geographical sample points were chosen randomly in proportion to the population size of the federal states and communities. In total, 120 sample points for GNHIES98, 180 sample points for DEGS1 and 160 sample points for KiGGS were included. Second, within each sample point persons were randomly selected stratified by age from local population registries.

#### GNHIES98

GNHIES98 was conducted from October 1997 to March 1999. It included a nationwide representative population-based sample of adults aged 18–79 years. The response rate was 61.4% [24, 25]. Overall, 7124 persons participated in GNHIES98. Of these, 4030 persons (2267 women, 1763 men) took part in the German Nutrition Survey 1998, a module of GNHIES98 [26]. The present analysis is restricted to participants of this substudy with valid 25(OH)D data (2211 women; 1706 men).

#### DEGS1

DEGS1 was conducted from November 2008 to December 2011. It included a nationwide representative population-based sample of adults aged 18–79 years. DEGS1 used a mixed design including both persons who already participated in the GNHIES98 (response rate 62%) and participants who were newly recruited by two-stage stratified random sampling (response rate 42%) [27]. Overall, 7987 adults participated in DEGS1 including 6995 persons with available serum 25(OH)D levels (3635 women; 3360 men).

#### KiGGS

KiGGS was conducted from May 2003 to May 2006 and included a nationwide representative population-based sample of children and adolescents aged 0–17 years. A total of 17,641 children and adolescents participated in KiGGS (response rate 66.6%) [30]. In the present study, we had to exclude 935 children < 1 year of age from whom no blood samples were obtained, 2319 children whose parents declined blood draw and 4366 participants whose blood was measured during the first study year before a change in laboratory method. Thus, in KiGGS we examined 10,015 participants with available serum 25(OH)D levels (4907 girls; 5108 boys).

### Data collection and laboratory measurement of serum 25-hydroxyvitamin D

All surveys comprised, among other survey instruments, measurements in blood samples. Venous blood samples were drawn at study centers and immediately processed and separated. Serum samples were aliquoted, stored at − 40 °C and then transported and analyzed at the central epidemiology laboratory unit at the Robert Koch Institute. Measurement of serum 25(OH)D was carried out using a chemiluminescent immunoassay (CLIA, LIAISON® 25 OH Vitamin D TOTAL Assay), one of the most commonly used methods in clinical and research laboratories. Details on analyses have been described elsewhere [20–22].

### Application of VDSP standardization protocol to existing 25(OH)D levels

As part of the European Commission–funded project ‘Food-based solutions for optimal vitamin D nutrition and health through the life cycle’ (ODIN), serum 25(OH)D levels from GNHIES, DEGS1 and KiGGS were retrospectively standardized by applying a VDSP protocol for standardization of existing serum 25(OH)D data [23]. The protocol has been described in detail elsewhere [11]. In brief, this included the 25(OH)D re-analysis of a subset (GNHIES98 *n* = 171; DEGS1 *n* = 163; KiGGS *n* = 160) of bio-banked serum samples (stored at − 40 °C) which were identified by dividing the range of the previous CLIA-based serum 25(OH)D measurements from the entire survey sample into quartiles, with each quartile being sampled according to a uniform distribution [19, 31]. The bio-banked serum samples from each of the studies were analyzed separately by using University College Cork’s LC-MS/MS assay, which has been certified by the Centers for Disease Control and Prevention (CDC) as being traceable to the Reference Measurement Procedures (RMP) of the National Institute for Standards and Technology (NIST), Ghent University, and CDC [9, 32–35].

### Statistical analyses

Analyses were performed with SPSS statistical software (version 20.0; SPSS, Chicago, IL, USA), SAS (version 9.4; SAS Institute, Cary, NC, USA) and STATA (version 12; StataCorp LP, College Station, TX, USA). Simple linear, piecewise linear and Deming regression models were used to examine the best-fit relation between serum 25(OH)D levels derived from CLIA and LC-MS/MS, which is described in detail elsewhere [16, 23]. The resulting regression equation which provided the best fit was applied to the entire data set from each study to create standardized data sets. Mean values and prevalence estimates of vitamin D deficiency, insufficiency and sufficiency (defined as serum 25(OH)D levels < 30 nmol/l, 30- < 50 nmol/l and > =50 nmol/l, respectively [3]) were calculated by sex and age groups based on original and standardized 25(OH)D data. For these analyses, a weighting factor was applied which adjusts for different sampling probabilities within the design strata and corrects deviations in the sample from the German population structure (at the time of each particular survey), taking into account age, sex, region, nationality, community type and education. The relation between the original and re-analyzed serum 25(OH)D values was analyzed using regression models (ordinary least squares, Deming, and piecewise), as described in detail elsewhere [16–19, 23].

## Results

Characteristics of the three study populations, stratified by sex and age group are shown in Table 1. The weighted percentage for sex and age groups reflect the distribution in the German population at time of survey. In GNHIES98, overall, 51.6% of the study population were women and 48.4% were men. In DEGS1, 50.2% of the participants were women, 49.8% were men. In KiGGS, 48.6% were girls and 51.4% were boys.

**Table 1:** Characteristics of the study populations of GNHIES98, DEGS1 and KiGGS^a,b^

		GNHIES98						DEGS1						KiGGS					
		Women		Men		Total		Women		Men		Total		Girls		Boys		Total	
		*n*	%	*n*	%	*n*	%	*n*	%	*n*	%	*n*	%	*n*	%	*n*	%	*n*	%
Age group (in years)																			
Adults	Children																		
18–29	1–2	440	17.1	328	18.8	768	17.9	534	18.4	513	19.2	1047	18.8	390	10.1	395	10.1	785	10.1
30–39	3–6	539	20.9	399	22.8	938	21.8	426	14.4	403	14.9	829	14.6	1050	21.9	1079	21.9	2129	21.9
40–49	7–10	425	17.5	301	19.0	726	18.2	683	20.9	593	22.1	1276	21.5	1274	22.7	1340	22.5	2614	22.6
50–59	11–13	376	16.8	312	17.5	688	17.1	744	18.3	636	18.4	1380	18.3	981	18.1	1047	17.9	2028	18.0
60–69	14–17	287	15.3	259	14.7	546	15.0	709	14.4	668	13.8	1377	14.1	1212	27.3	1247	27.5	2459	27.4
70–79	–	144	12.3	107	7.3	251	9.9	539	13.6	547	11.6	1086	12.6	–	–	–	–	–	–
Total		2211	51.6	1706	48.4	3917	100.0	3635	50.2	3360	49.8	6995	100.0	4907	48.6	5108	51.4	10,015	100.0

^a^ Results are weighted, except the number of cases

^b^ Rounding may result in minor variations in totals and percentages

**Fig. 1:**
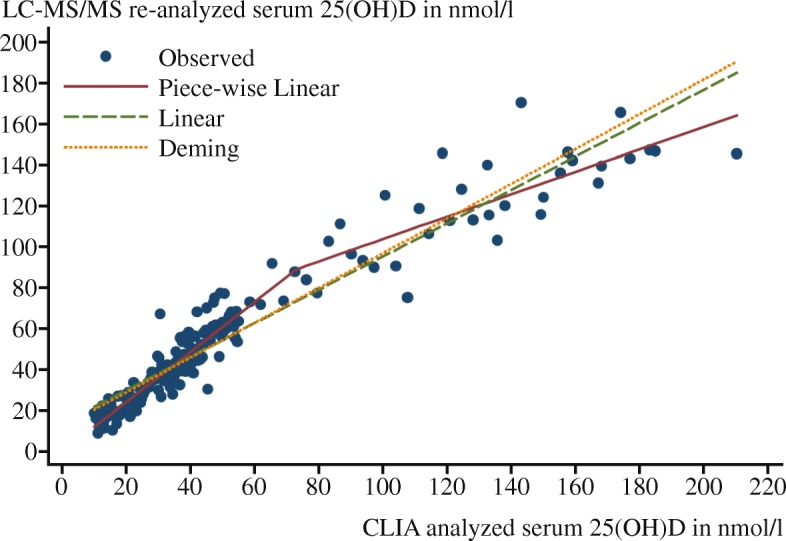
Calibration study results used to standardize serum samples from GNHIES98. 25(OH)D, Serum 25-hydroxyvitamin D; CLIA, Chemiluminescent immunoassay; LC-MS/MS, Liquid chromatography-tandem mass spectrometry; GNHIES98, German National Health Interview and Examination Survey 1998

**Fig. 2:**
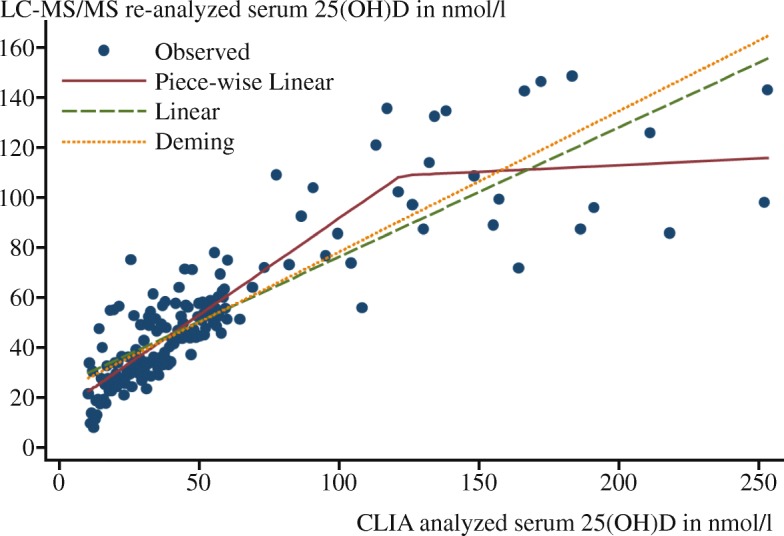
Calibration study results used to standardize serum samples from DEGS1. 25(OH)D, Serum 25-hydroxyvitamin D; CLIA, Chemiluminescent immunoassay; LC-MS/MS, Liquid chromatography-tandem mass spectrometry; DEGS1, German Health Interview and Examination Survey for Adults

**Fig. 3:**
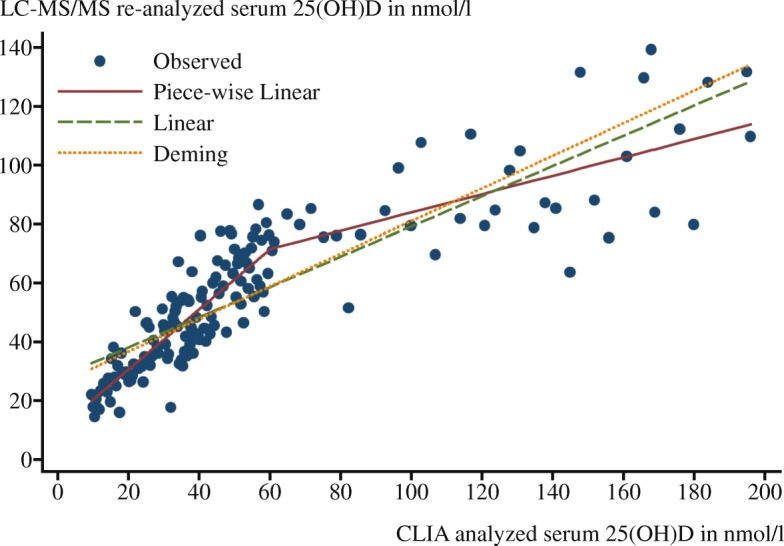
Calibration study results used to standardize serum samples from KiGGS. 25(OH)D, Serum 25-hydroxyvitamin D; CLIA, Chemiluminescent immunoassay; LC-MS/MS, Liquid chromatography-tandem mass spectrometry; KiGGS, German Health Interview and Examination Survey for Children and Adolescents

The coefficients of the regression equations describing the relation between 25(OH)D in the VDSP identified subsets of serum samples from GNHIES98, DEGS1 and KiGGS, originally measured by CLIA and re-analyzed by LC-MS/MS, is shown in Table 2. For all three study populations, piecewise regression models provided the best fit (Figs. 1, 2, and 3) and the resulting regression equations were applied to the entire data set from each of the respective studies. The change points for the piecewise regression lines were about 73 for GNHIES98, 122 for DEGS1 and 60.5 for KiGGS samples, respectively (Table 2). For the GNHIES98 and KIGGS samples the lines are still rising after these points but less steeper. For DEGS1 it becomes almost flat.

**Table 2:** Coefficients of the piecewise regression model between 25(OH)D levels derived from CLIA and LC-MS/MS

	If Rval^a^ ≤ value			If Rval ≥ value			
Study	Value	Intercept 1	X1^b^	Value	Intercept 2	X2^b^	R^2^
GNHIES98	73.2698	−0.2256	1.2197	73.2698	49.0336	0.5474	0.94
DEGS1	121.9968	14.5310	0.7715	121.9968	102.1919	0.0530	0.79
KiGGS	60.5211	9.4005	1.0225	60.5211	52.4099	0.3119	0.81

*25(OH)D* Serum 25-hydroxyvitamin D, *CLIA* Chemiluminescent immunoassay, *LC-MS/MS* Liquid chromatography-tandem mass spectrometry, *GNHIES98* German National Health Interview and Examination Survey 1998, *DEGS1* German Health Interview and Examination Survey for Adults, *KiGGS* German Health Interview and Examination Survey for Children and Adolescents

^a^ In piecewise regression, the independent variable is partitioned into intervals and a separate line segment is fit to each interval (Rval = change point in serum 25(OH)D concentration) [23]

^b^ X1, X2 = slopes of the regression lines

**Fig. 4:**
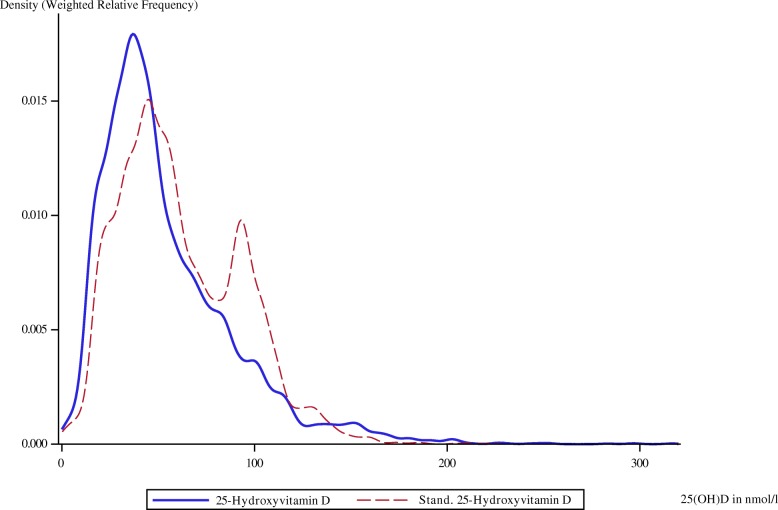
Weighted relative frequency for original and standardized serum 25(OH)D of GNHIES98 samples. 25(OH)D, Serum 25-hydroxyvitamin D; GNHIES98, German National Health Interview and Examination Survey 1998

**Fig. 5:**
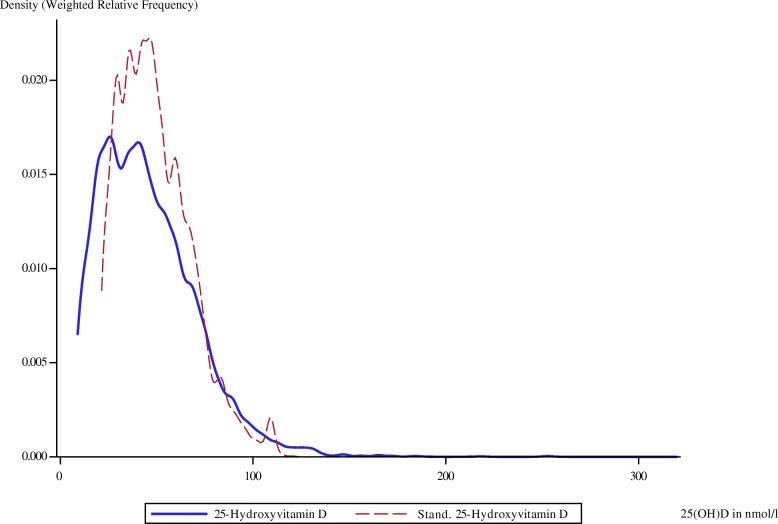
Weighted relative frequency for original and standardized serum 25(OH)D of DEGS1 samples. 25(OH)D, Serum 25-hydroxyvitamin D; DEGS1, German Health Interview and Examination Survey for Adults

**Fig. 6:**
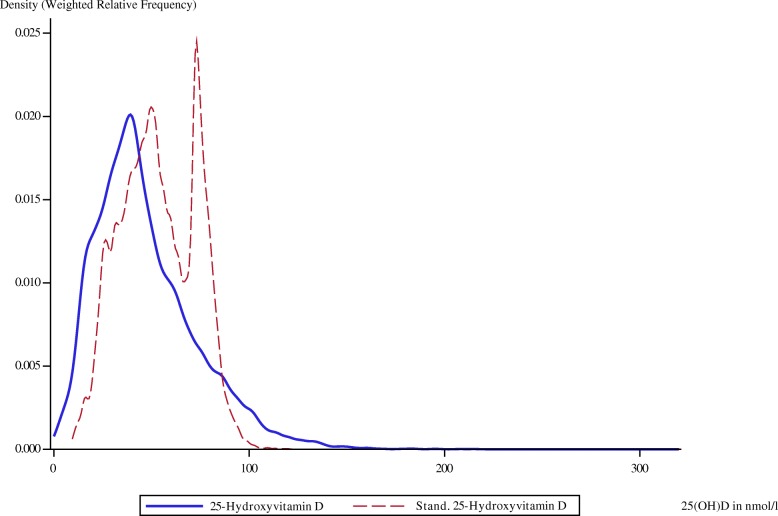
Weighted relative frequency for original and standardized serum 25(OH)D of KiGGS samples. 25(OH)D, Serum 25-hydroxyvitamin D; KiGGS, German Health Interview and Examination Survey for Children and Adolescents

The weighted relative frequencies for serum 25(OH)D as measured by using the CLIA and after standardization with the regression equations derived from the comparison with LC–MS/MS are shown in Figs. 4, 5, and 6. The distinct peaks are due to the underlying piecewise regression models. In each survey, standardization shifted the distribution of 25(OH)D to the right.

Moreover, in each survey, mean serum 25(OH)D levels based on the original measurements were lower in comparison to measurements after standardization across all age groups in men and women (Tables 3 and 4) and in girls and boys (Table 5). In GNHIES98, the standardized mean serum 25(OH)D level was 62.0 nmol/l (58.9–65.1) in women and 60.9 nmol/l (57.1–64.7) in men (Table 3), whereas the standardized mean serum 25(OH)D level in DEGS1 was 49.7 nmol/l (48.2–51.3) among women and 49.3 nmol/l (47.4–51.2) among men (Table 4). In KiGGS, the standardized mean serum 25(OH)D level was 53.2 nmol/l (51.1–55.3) in girls and 53.7 nmol/l (51.5–56.0) in boys (Table 5).

**Table 3:** Means and prevalence of 25(OH)D categories based on original and standardized 25(OH)D levels of GNHIES98^a^

	Mean serum 25(OH)D levels		Serum 25(OH)D levels by thresholds of the IOM 2011 in %					
			< 30 nmol/l		30- < 50 nmol/l		> = 50 nmol/l	
	Original	Standardized	Original	Standardized	Original	Standardized	Original	Standardized
	nmol/l (95% CI)	nmol/l (95% CI)	% (95% CI)	% (95% CI)	% (95% CI)	% (95% CI)	% (95% CI)	% (95% CI)
Women	55.4 (51.8–59.0)	62.0 (58.9–65.1)	23.8 (20.2–27.8)	15.8 (12.8–19.3)	34.0 (30.8–37.3)	28.0 (25.2–30.9)	42.2 (38.3–46.2)	56.3 (52.3–60.2)
18–29 years	65.4 (59.5–71.4)	70.8 (65.7–75.8)	19.5 (14.4–25.9)	18.7 (12.9–26.2)	26.5 (21.8–31.8)	23.7 (18.9–29.3)	54.0 (47.3–60.5)	57.6 (50.3–64.6)
30–39 years	60.3 (55.4–65.2)	66.6 (62.7–70.5)	19.0 (15.2–23.4)	14.4 (10.6–19.4)	32.4 (28.1–37.0)	26.6 (22.2–31.6)	48.6 (47.3–53.7)	58.9 (52.3–65.3)
40–49 years	57.5 (52.8–62.3)	64.4 (60.4–68.4)	21.2 (16.6–26.7)	14.9 (10.5–20.7)	32.5 (27.3–38.2)	30.5 (24.8–36.9)	46.3 (40.5–52.1)	54.6 (46.6–62.4)
50–59 years	52.9 (47.4–58.4)	60.2 (55.4–65.1)	24.2 (18.8–30.5)	17.8 (13.3–23.4)	36.1 (30.5–42.1)	27.4 (22.1–33.4)	39.7 (33.5–46.2)	54.8 (47.5–61.9)
60–69 years	49.3 (44.4–54.2)	56.5 (52.0–61.1)	26.7 (20.1–34.5)	7.3 (4.4–11.8)	40.9 (34.1–48.2)	23.9 (18.3–30.6)	32.4 (26.0–39.5)	68.8 (61.3–75.4)
70–79 years	40.9 (35.0–46.9)	48.2 (42.3–54.1)	37.4 (27.4–48.7)	18.5 (10.7–29.9)	37.7 (28.2–48.3)	31.7 (21.4–44.1)	24.9 (16.3–36.0)	49.9 (37.5–62.3)
Men	53.4 (49.1–57.6)	60.9 (57.1–64.7)	23.6 (19.8–28.0)	15.1 (12.2–18.7)	33.2 (29.9–36.5)	26.9 (23.7–30.5)	43.2 (38.1–48.5)	57.9 (52.7–62.9)
18–29 years	52.2 (47.5–56.8)	60.1 (55.5–64.7)	23.3 (16.9–31.1)	11.9 (8.4–16.7)	31.4 (25.6–37.9)	19.8 (15.5–25.0)	45.3 (38.0–52.8)	68.3 (61.5–74.4)
30–39 years	55.2 (50.0–60.3)	62.3 (57.8–66.9)	22.9 (17.8–28.9)	11.7 (8.7–15.6)	33.4 (28.9–38.3)	27.3 (24.0–31.0)	43.7 (37.8–49.8)	60.9 (56.3–65.4)
40–49 years	51.5 (44.9–58.1)	59.2 (53.2–65.3)	25.4 (19.3–32.5)	13.1 (9.5–17.9)	34.4 (28.0–41.5)	27.7 (23.5–32.3)	40.2 (32.0–49.1)	59.2 (53.8–64.4)
50–59 years	49.4 (44.4–54.4)	57.1 (52.4–61.9)	26.8 (21.4–33.1)	16.0 (11.7–21.4)	34.7 (29.0–40.9)	28.2 (22.7–34.4)	38.5 (31.3–46.2)	55.8 (48.9–62.2)
60–69 years	60.3 (54.2–66.5)	67.7 (62.5–72.8)	15.3 (10.7–21.4)	16.9 (11.7–23.8)	33.9 (27.5–40.9)	33.8 (27.9–40.4)	50.8 (42.9–58.7)	49.2 (42.3–56.1)
70–79 years	51.1 (43.3–58.9)	58.4 (50.8–66.0)	31.3 (21.0–44.0)	30.2 (20.3–42.3)	28.2 (18.3–40.8)	33.0 (24.0–43.5)	40.4 (29.2–52.8)	36.9 (27.0–47.9)
Total	54.4 (50.8–58.0)	61.5 (58.3–64.7)	23.7 (20.3–27.5)	15.5 (12.8–18.6)	33.6 (31.0–36.3)	27.5 (25.1–30.0)	42.7 (38.7–46.8)	57.1 (53.0–61.1)

*25(OH)D* Serum 25-hydroxyvitamin D, *GNHIES98* German National Health Interview and Examination Survey 1998, *IOM* Institute of Medicine, USA, *CI* confidence interval

^a^ Results are weighted population estimates

**Table 4:** Means and prevalence of 25(OH)D categories based on original and standardized 25(OH)D levels of DEGS1^a^

	Mean serum 25(OH)D levels		Serum 25(OH)D levels by thresholds of the IOM 2011 in %					
			< 30 nmol/l		30- < 50 nmol/l		> = 50 nmol/l	
	Original	Standardized	Original	Standardized	Original	Standardized	Original	Standardized
	nmol/l (95% CI)	nmol/l (95% CI)	% (95% CI)	% (95% CI)	% (95% CI)	% (95% CI)	% (95% CI)	% (95% CI)
Women	45.9 (43.8–47.9)	49.7 (48.2–51.3)	29.7 (26.5–33.1)	14.7 (12.5–17.2)	31.8 (29.7–33.9)	41.0 (38.5–43.6)	38.6 (35.0–42.3)	44.3 (40.6–48.1)
18–29 years	52.4 (49.2–55.7)	54.2 (51.9–56.5)	25.1 (20.7–30.0)	14.4 (10.7–19.1)	28.4 (24.0–33.3)	34.6 (29.7–39.8)	46.5 (40.8–52.2)	51.0 (45.3–56.7)
30–39 years	48.2 (43.9–52.4)	51.5 (48.3–54.7)	32.2 (26.1–39.0)	18.7 (14.1–24.4)	24.4 (20.1–29.2)	33.0 (28.1–38.3)	43.4 (36.7–50.4)	48.3 (41.3–55.3)
40–49 years	46.1 (43.0–49.2)	50.0 (47.6–52.3)	29.5 (24.4–35.2)	14.8 (11.2–19.2)	30.7 (26.6–35.3)	39.3 (34.7–44.1)	39.8 (34.4–45.4)	45.9 (40.2–51.7)
50–59 years	43.5 (41.2–45.7)	48.1 (46.3–49.8)	30.3 (25.8–35.1)	14.9 (11.6–18.8)	32.5 (28.4–36.9)	42.2 (37.9–46.7)	37.2 (32.1–42.6)	42.9 (37.7–48.3)
60–69 years	43.9 (41.5–46.4)	48.4 (46.5–50.3)	27.2 (22.9–31.9)	9.8 (7.4–12.9)	36.9 (32.2–41.7)	48.5 (43.3–53.7)	36.0 (30.4–41.9)	41.7 (36.2–47.6)
70–79 years	39.8 (37.3–42.2)	45.2 (43.3–47.1)	35.4 (29.4–41.8)	15.9 (12.0–20.7)	39.2 (33.9–44.8)	51.1 (45.2–57.0)	25.5 (20.5–31.2)	33.0 (27.5–39.0)
Men	45.3 (42.8–47.8)	49.3 (47.4–51.2)	30.8 (26.8–35.2)	15.7 (12.9–19.0)	30.9 (28.4–33.6)	40.5 (37.3–43.8)	38.3 (33.8–42.9)	43.7 (39.2–48.4)
18–29 years	46.7 (42.7–50.8)	50.5 (47.4–53.5)	31.6 (26.1–37.6)	16.2 (12.2–21.1)	29.1 (24.6–34.1)	39.7 (34.3–45.4)	39.3 (33.3–45.7)	44.1 (37.9–50.4)
30–39 years	43.4 (39.3–47.5)	48.0 (44.8–51.2)	36.4 (29.5–43.9)	19.0 (14.0–25.2)	28.9 (23.6–35.0)	42.7 (35.4–50.2)	34.7 (27.6–42.4)	38.4 (31.0–46.3)
40–49 years	45.2 (41.7–48.8)	49.0 (46.5–51.6)	33.6 (27.4–40.3)	19.4 (14.4–25.5)	26.6 (21.9–32.0)	36.0 (30.5–41.9)	39.8 (33.6–46.4)	44.6 (38.1–51.4)
50–59 years	44.2 (41.7–46.7)	48.6 (46.7–50.5)	29.5 (24.3–35.2)	13.3 (9.6–18.1)	34.4 (30.0–39.2)	44.9 (40.0–49.9)	36.1 (30.8–41.7)	41.8 (36.5–47.3)
60–69 years	48.0 (45.0–50.9)	51.3 (49.1–53.6)	22.1 (17.2–27.9)	9.9 (7.0–13.8)	33.3 (28.1–38.9)	38.1 (32.8–43.6)	44.6 (38.3–51.2)	52.1 (45.7–58.4)
70–79 years	43.9 (40.9–46.9)	48.3 (46.1–50.6)	29.7 (23.7–36.5)	14.9 (10.5–20.8)	36.3 (30.7–42.3)	43.6 (37.7–49.7)	34.0 (28.1–40.5)	41.5 (34.9–48.4)
Total	45.6 (43.5–47.7)	49.5 (47.9–51.1)	30.2 (26.9–33.8)	15.2 (13.0–17.8)	31.3 (29.4–33.3)	40.8 (38.3–43.3)	38.4 (34.7–42.3)	44.0 (40.2–47.9)

*25(OH)D* Serum 25-hydroxyvitamin D, *DEGS1* German Health Interview and Examination Survey for Adults, *IOM* Institute of Medicine, USA, *CI* confidence interval

^a^ Results are weighted population estimates

**Table 5:** Means and prevalence of 25(OH)D categories based on original and standardized 25(OH)D levels of KiGGS^a^

	Mean serum 25(OH)D levels		Serum 25(OH)D levels by thresholds of the IOM 2011 in %					
			< 30 nmol/l		30- < 50 nmol/l		> = 50 nmol/l	
	Original	Standardized	Original	Standardized	Original	Standardized	Original	Standardized
	nmol/l (95% CI)	nmol/l (95% CI)	% (95% CI)	% (95% CI)	% (95% CI)	% (95% CI)	% (95% CI)	% (95% CI)
Girls	46.7 (44.1–49.3)	53.2 (51.1–55.3)	27.1 (22.9–31.9)	12.5 (10.0–15.7)	36.8 (34.4–39.2)	33.5 (30.3–36.8)	36.1 (31.8–40.6)	54.0 (48.9–59.0)
1–2 years	62.1 (58.5–65.7)	63.8 (61.3–66.3)	14.2 (10.4–19.0)	5.7 (3.5–9.0)	24.7 (19.6–30.6)	19.7 (15.2–25.2)	61.1 (53.9–67.9)	74.6 (68.5–79.9)
3–6 years	48.8 (45.6–52.0)	55.3 (52.9–57.7)	22.2 (17.7–27.4)	9.1 (6.5–12.6)	38.5 (34.9–42.2)	31.8 (27.6–36.3)	39.4 (33.9–45.1)	59.1 (52.9–65.0)
7–10 years	43.5 (40.8–46.3)	51.4 (49.1–53.7)	28.3 (23.2–34.0)	12.2 (9.2–16.1)	40.2 (36.4–44.2)	36.7 (32.2–41.3)	31.5 (26.4–37.1)	51.1 (44.9–57.3)
11–13 years	37.8 (35.3–40.3)	46.7 (44.4–49.0)	36.2 (30.3–42.5)	18.9 (14.7–24.1)	41.3 (37.1–45.6)	41.4 (37.3–45.7)	22.6 (18.4–27.3)	39.6 (34.0–45.5)
14–17 years	48.0 (44.8–51.1)	53.5 (51.1–56.0)	29.0 (24.1–34.5)	13.9 (10.8–17.6)	34.0 (30.8–37.3)	32.0 (28.4–35.7)	37.0 (32.1–42.2)	54.2 (48.6–59.7)
Boys	47.4 (44.6–50.2)	53.7 (51.5–56.0)	26.9 (22.5–31.7)	12.5 (9.7–15.8)	35.3 (33.1–37.6)	32.7 (29.4–36.2)	37.8 (33.3–42.6)	54.8 (49.4–60.1)
1–2 years	66.2 (62.2–70.2)	66.3 (63.9–68.8)	10.8 (7.8–14.9)	4.9 (2.8–8.2)	23.0 (18.5–28.2)	15.5 (11.6–20.5)	66.2 (59.8–72.0)	79.6 (73.7–84.5)
3–6 years	48.4 (44.9–51.8)	54.6 (51.9–57.3)	26.4 (21.1–32.4)	11.5 (8.4–15.5)	32.6 (29.0–36.3)	31.5 (27.0–36.3)	41.1 (35.2–47.2)	57.0 (50.4–63.5)
7–10 years	46.4 (43.5–49.4)	53.8 (51.4–56.3)	23.8 (19.1–29.2)	10.9 (8.0–14.6)	39.4 (35.9–43.0)	31.6 (27.2–36.3)	36.8 (31.3–42.7)	57.5 (51.1–63.8)
11–13 years	45.0 (41.5–48.5)	52.1 (49.6–54.6)	26.9 (22.1–32.3)	11.0 (7.8–15.3)	41.9 (38.5–45.5)	39.2 (34.1–44.5)	31.2 (26.3–36.5)	49.8 (43.5–56.1)
14–17 years	42.1 (39.1–45.0)	49.4 (47.0–51.9)	35.7 (30.2–41.6)	18.3 (14.4–22.9)	34.3 (30.8–38.0)	36.8 (33.3–40.4)	30.0 (25.5–34.9)	44.9 (39.4–50.7)
Total	47.1 (44.4–49.7)	53.5 (51.3–55.6)	27.0 (22.8–31.7)	12.5 (9.9–15.6)	36.0 (34.0–38.1)	33.1 (30.0–36.3)	37.0 (32.7–41.5)	54.4 (49.3–59.5)

*25(OH)D* Serum 25-hydroxyvitamin D, *KiGGS* German Health Interview and Examination Survey for Children and Adolescents, *IOM* Institute of Medicine, USA, *CI* confidence interval

^a^ Results are weighted population estimates

**Fig. 7:**
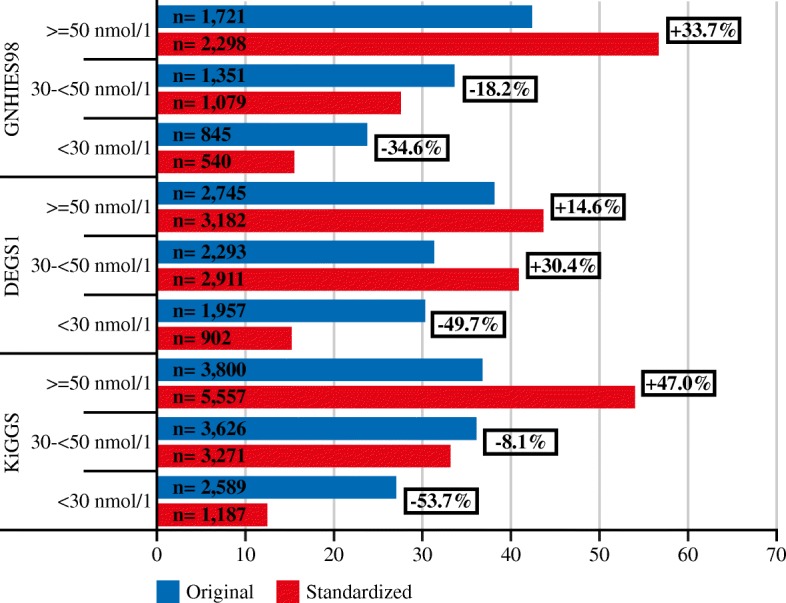
25(OH)D categories based on original and standardized serum 25(OH)D levels of GNHIES98, DEGS1 and KiGGS. 25(OH)D, Serum 25-hydroxyvitamin D; GNHIES98, German National Health Interview and Examination Survey 1998; DEGS1, German Health Interview and Examination Survey for Adults; KiGGS, German Health Interview and Examination Survey for Children and Adolescents

Following standardization of serum 25(OH)D data, the estimated prevalence of vitamin D deficiency (i.e. serum 25(OH)D < 30 nmol/l) in the population was lowered by half in both DEGS1 (from 30.2 to 15.2%) and KiGGS (from 27.0 to 12.5%) and by one third in GNHIES98 (from 23.7 to 15.5%) (Tables 3, 4, and 5 and Fig. 7). In contrast, the estimated prevalence of vitamin D sufficiency (i.e. serum 25(OH)D > =50 nmol/l) increased after standardization in each of the surveys (Tables 3, 4, and 5 and Fig. 7).

The effects of standardization are somewhat different for specific sex and age groups. Subgroup analyses also clearly show differences between analyses of originally measured and standardized values, especially in the low and high end of the distribution (Tables 3, 4, and 5). This is due to the fact that the CLIA used in GNHIES98 and KiGGS underestimated serum 25(OH)D in the low end of the distribution and overestimated it in the high end whereas the CLIA used in DEGS1, however, underestimated serum 25(OH)D in both the low and high end of the distribution (Table 6).

**Table 6:** Predicted data shifts from original to standardized 25(OH)D levels for GNHIES98, DEGS1 and KiGGS

GNHIES98		DEGS1		KiGGS	
Serum 25(OH)D levels in nmol/l		Serum 25(OH)D levels in nmol/l		Serum 25(OH)D levels in nmol/l	
Original	Standardized	Original	Standardized	Original	Standardized
1	1	1	15	1	10
5	6	5	18	5	15
10	12	10	22	10	20
15	18	15	26	15	25
20	24	20	30	20	30
25	30	25	34	25	35
30	36	30	38	30	40
40	49	40	45	40	50
50	61	50	53	50	61
60	73	60	61	60	71
75	91	75	72	75	76
80	93	80	76	80	77
90	98	90	84	90	80
100	104	100	92	100	84
125	117	125	109	125	91
150	131	150	110	150	99
200	159	200	113	200	115
250	186	250	115	250	130
300	213	300	118	300	146

*25(OH)D* Serum 25-hydroxyvitamin D, *GNHIES98* German National Health Interview and Examination Survey 1998, *DEGS1* German Health Interview and Examination Survey for Adults, *KiGGS* German Health Interview and Examination Survey for Children and Adolescents

In GNHIES98, the proportion of serum 25(OH)D< 30 nmol/l was higher in women aged 18 to 29 than in men of the same age (18.7% vs. 11.9%), while the proportion of serum 25(OH)D > 50 nmol/l was higher in men aged 18 to 29 years than in women of the same age (68.3% vs. 57.6%) (Table 3). However, men aged 60 to 69 years and 70 to 79 years had proportions of serum levels of 25(OH)D < 30 nmol/l which were twice as high as those of women of the same age (60 to 69 years: 16.9% vs. 7.3%; 70 to 79 years: 30.2% vs. 18.5%). At the other side, the proportion of serum 25(OH)D levels > 50 nmol/l was much higher in women aged 60 to 69 years and 70 to 79 years than in men (60 to 69 years: 68.8% vs. 49.2%; 70 to 79 years: 49.9% vs. 36.9%).

In DEGS1, the proportion of standardized serum 25(OH)D values < 30 nmol/l was relatively stable across all age groups and sexes, ranging from 13.3 to 19.4% (Table 4). Only participants aged 60 to 69 showed markedly lower proportions with 9.8% among women and 9.9% among men. Women aged 30 to 39 years had a higher proportion of serum 25(OH)D values > 50 nmol/l than men of the same age (48.3% vs. 38.4%), while the opposite was true for the two highest age groups (60 to 69 years: 52.1% vs. 41.7%; 70 to 79 years: 41.5% vs. 33.0%).

In KiGGS, the prevalence of vitamin D deficiency increased from young children to teenagers (Table 5). In children aged 1 to 2 years, the proportion of serum 25(OH)D < 30 nmol/l was about 5% in both girls and boys and the proportion of serum 25(OH)D > 50 nmol/l was about 75% each. In girls aged 11 to 13 years, however, 18.9% had 25(OH)D levels < 30 nmol/l which was higher than the 11.0% of boys of the same age (Table 5). In return, boys aged 11 to 13 years had higher proportions of serum 25(OH)D levels > 50 nmol/l than girls of the same age (49.8% vs. 39.6%). In contrast, in the age group 14 to 17 years, girls had a lower proportion of 25(OH)D levels < 30 nmol/l (13.9%) than boys of the same age (18.3%), while boys aged 14 to 17 years had lower proportions of serum 25(OH)D levels> 50 nmol/l than girls of the same age (44.9% vs. 54.2%).

A comparison of the prevalence of vitamin D deficiency, insufficiency (i.e. serum 25(OH)D > 30 but < 50 nmol/l) and sufficiency in GNHIES98 and DEGS1 using the standardized serum 25(OH)D data provides an insight into temporal changes in vitamin D status in the adult German population over a decade. The prevalence of vitamin D deficiency in all adults was very similar in both surveys(~ 15%), but the prevalence of insufficiency was much higher in the more recent DEGS1 survey than GNHIES98 (41% vs. 27%, respectively) (Tables 3 and 4). Likewise, the prevalence of sufficiency was lower in DEGS1 than GNHIES98 (44% vs. 57%, respectively).

## Discussion

The present work highlights how standardization of 25(OH)D data has a substantial impact on estimates of the vitamin D status in Germany including higher mean levels, higher prevalence of vitamin D sufficiency and lower prevalence of vitamin D deficiency overall as well as in age- and sex-specific analyses. Although the proportion of persons with deficient 25(OH)D serum levels is substantially smaller than originally reported, it is still a considerable number of people within the German population. For example, using the prevalences of vitamin D deficiency based on the new standardized data on serum 25(OH)D < 30 nmol/l from DEGS1 and KiGGS, about 15% of adults and 12.5% of children were vitamin D deficient which amounts to 11 million persons within the German population. It is also clear from the standardized serum 25(OH)D data from GNHIES98 and DEGS1 in the present work that the prevalence of vitamin D deficiency has remained stable over a ten year period. However, the prevalence of insufficiency (30- < 50 nmol/l) was much higher in the more recent DEGS1 survey than GNHIES98 (41% vs. 27%, respectively). The present work did not seek to explore potential underpinning reasons for changes in vitamin D status over time in the German adult population, but this difference may be related to changes in outdoor activity, sun tan behavior or sunscreen use [36].

Within all of the three surveys blood samples were taken on a voluntary base. We assume that this procedure did not cause a systematic selection bias, however, also a weighting factor was used to correct for deviations compared to the population structure at the time of each of the surveys. Within GNHIES98, serum 25(OH)D was measured in a subsample which also participated in a nutrition module. Participation was randomized; however, women of child bearing age were oversampled because of a connected folate study. This deviation in the sample was corrected by using a specific weighting factor.

The new data on three German national health surveys complement and extend data from several surveys either side of the Atlantic that have been standardized according to VDSP protocols in recent years [16, 17, 19, 23]. Collectively, all of these exercises clearly show that the originally used assays demonstrate varying precision across the entire measuring range resulting in minor to major differences (both positive and negative) between original and standardized serum 25(OH)D data. This was even the case for studies using the same kind of assay, e.g. CLIA (the original assay used in the three German surveys), as demonstrated recently in the data from the ODIN project [23]. For example, in an Icelandic cohort study including 5519 adults with mean age of 77 years, the prevalence of serum 25(OH)D level < 30 nmol/l (indicating deficiency) was lower at 8.4% after standardization compared to the original estimate of 17.2%. In a population-based survey conducted in the UK with 977 participants aged > = 19 years, prevalence of deficiency was about a fifth lower after standardization (30.7% vs. 24.0%). However, in a Dutch cohort study including 915 women aged > = 55 years prevalence estimates for serum 25(OH)D levels < 30 nmol/l were slightly higher after standardization (3.8% vs. 4.6%).

Likewise, divergent results in terms of the impact of standardization have also been reported in surveys for children and adolescents, which have used the CLIA [23]. Baseline serum 25(OH)D data from a cross-over trial conducted in Denmark including 779 children aged 8–11 years, for example, found a higher prevalence of 25(OH)D levels < 30 nmol/l (6.2% compared to 5.0%), whereas a population-based survey from the UK with 511 children and adolescents aged 1–18 years described a moderately lower prevalence compared to original values (18.4% versus 23.3%).

Besides the well-reported between-laboratory differences even for the same assay [14], these differences may also be linked to methodological issues even within the CLIA assay over time e.g. assay drift/shift or changes in assay composition like reformulation of reagents [37, 38]. It is possible that the process of standardization of the German data may have contributed to the observed differences. First, the storage time of re-analyzed blood samples used for VDSP standardization was relatively long (4–17 years). However, several studies indicate that serum 25(OH)D is stable even after long-term storage [39–42]. Moreover, multiple freeze-thaw cycles also seem to have no considerable consequences on serum 25(OH)D [43, 44]. Second, analyses and re-analyses were conducted in different laboratories and as mentioned above, this might have had an effect on differences seen in original and standardized 25(OH)D data. Third, only 160–171 bio-banked blood samples were re-analyzed with LC-MS/MS to develop a calibration equation to predict 25(OH)D levels for the entire samples of each survey. However, these 160–171 samples were based on power calculations and also were derived from a specific uniform sampling procedure within quartiles which has been shown in simulations to provide for the most efficient coverage of the full distribution of the sample in question. In addition, previous studies showed very good concordance between VDSP protocol predicted 25(OH)D levels and analyses comprising the entire sample [16].

Challenges in the evaluation and comparison of vitamin D data may also be caused by the fact that there is currently no consensus on optimal levels of serum 25(OH)D [3, 4, 45, 46]. In addition, the most commonly used thresholds to define vitamin D deficiency, insufficiency and sufficiency (25(OH)D levels, < 30, 30- < 50 and> =50 nmol/l, respectively, as suggested by the IOM [3]), are being used irrespective from the assay employed.

According to the wide range of methodological issues, the interpretation of the actual vitamin D status is complicated, especially the diagnosis of vitamin D deficiency. Hence, in clinical settings, the potential misclassification of 25(OH)D levels may result in over- or undertreatment of patients and difficulties in monitoring supplementation therapy.

Furthermore, variable and non-comparable 25(OH)D levels also impede epidemiological research, e.g. the development and establishment of evidence-based reference values for the evaluation of vitamin D status, especially deficiency and sufficiency, is challenging [45].

For instance, data sources which have been used to derive reference values for Vitamin D deficiency are also largely based on unstandardized 25(OH)D measurements. The consequences of this are not totally clear and this derivation process should be revised [45]. Moreover, valid quantification of vitamin D deficiency in (nationally representative) populations is required to develop dietary reference values as well as to evaluate the need for evidence-based public health strategies e.g. food fortification [16, 23].

The current results show that, depending on the laboratory method, the estimation of population quantities at risk of deficiency and insufficiency may differ substantially which should be considered while implementing prevention measures. Accurate quantification of the magnitude of the public health problem is a critical piece of data upon which national health policy relies. It has been suggested that from a public health perspective, a prevalence of micronutrient deficiency at a rate greater than 20% in the entire population and/or in subsets of the population considered especially at risk (e.g., infants, children and pregnant women) constitutes a public health issue that may warrant intervention [47]. This is of consequence in the present work where the estimate of vitamin D deficiency was > 20% in all three German surveys before standardization, but all fell below 20% following standardization.

However, a previous publication of the ODIN (Food-based solutions for optimal vitamin D nutrition and health through the life cycle) project included sensitivity analyses on the impact of the standardization on estimates of the association between vitamin D and all-cause mortality as a major adverse outcome measure [48]. For the mortality risk estimates of pooled data analyses that included data from our surveys, the work reported that no major differences between original and standardized 25(OH)D concentrations were observed, but a few percent of the participants, which is relevant from a public health perspective, were indeed re-classified into different 25(OH)D groups after the standardization procedure. Estimates for the DEGS1 data in particular became slightly lower after standardization but did not differ significantly from results using original data. In general, the standardization had an important impact on classification of subgroups, but it has not a large impact on individual risk estimation.

## Conclusions

The use of standardization of 25(OH)D levels has a substantial impact on estimates of the vitamin D status in Germany. Clinical diagnostic and therapeutic as well as public health decision-making require valid and comparable data. Therefore, standardization and calibration of commercial, clinical and research laboratory assays for 25(OH)D measurement should become common practice. Although the VDSP made substantial progress to overcome the mentioned problems in the past few years, there is still some way to go. Until then, laboratory technicians, physicians, researchers, and authorities must be aware of limitations when comparing and interpreting vitamin D values especially those derived from different assays and laboratories.

Disclaimer: The findings and conclusions in this report are those of the authors and do not necessarily represent the views of the National Institutes of Health or the US Department of Health and Human Services.

## Abbreviations

25(OH)D: Serum 25-hydroxyvitamin D
CDC: Centers for Disease Control and Prevention
CLIA: Chemiluminescent immunoassay
DEGS1: German Health Interview and Examination Survey for Adults
GNHIES98: German National Health Interview and Examination Survey 1998
HPLC: High performance liquid chromatography
IOM: Institute of Medicine, USA
KiGGS: German Health Interview and Examination Survey for Children and Adolescents
LC-MS/MS: Liquid chromatography-tandem mass spectrometry
NIST: National Institute for Standards and Technology
ODIN: Food-based solutions for optimal vitamin D nutrition and health through the life cycle
RMP: Reference Measurement Procedures
VDSP: Vitamin D Standardization Program
